# Prevention of Typhoid Fever by Existing Improvements in Household Water, Sanitation, and Hygiene, and the Use of the Vi Polysaccharide Typhoid Vaccine in Poor Urban Slums: Results from a Cluster-Randomized Trial

**DOI:** 10.4269/ajtmh.21-1034

**Published:** 2022-03-07

**Authors:** Justin Im, Farhana Khanam, Faisal Ahmmed, Deok Ryun Kim, Sophie Kang, Birkneh Tilahun Tadesse, Fahima Chowdhury, Tasnuva Ahmed, Asma Binte Aziz, Masuma Hoque, Md. Taufiqul Islam, Juyeon Park, Xinxue Liu, Dipika Sur, Gideok Pak, Hyon Jin Jeon, Khalequ Zaman, Ashraful Islam Khan, Firdausi Qadri, Florian Marks, Jerome H. Kim, John D. Clemens

**Affiliations:** ^1^ International Vaccine Institute, Seoul, Republic of Korea;; ^2^ International Centre for Diarrheal Disease Research, Bangladesh, Dhaka, Bangladesh;; ^3^ Department of Medicine, University of Cambridge, Cambridge, United Kingdom;; ^4^ Oxford Vaccine Group, Department of Paediatrics, University of Oxford, Oxford, United Kingdom;; ^5^ National Institute of Cholera and Enteric Diseases, Kolkata, West Bengal, India;; ^6^ University of Antananarivo, Antananarivo, Madagascar;; ^7^ University of California Los Angeles Fielding School of Public Health, Los Angeles, California

## Abstract

Modest improvements in household water, sanitation, and hygiene (WASH) and typhoid vaccination can reduce typhoid risk in endemic settings. However, empiric evaluation of their combined impact is lacking. A total of 62,756 persons residing in 80 clusters in a Kolkata slum were allocated randomly 1:1 to either the typhoid Vi polysaccharide (ViPS) vaccine or hepatitis A (Hep A) vaccine. Surveillance was conducted for 2 years before and 2 years after vaccination. We classified households as having “better” or “not better” WASH, and calculated the prevalence of better WASH households in clusters using previously validated criteria. We evaluated the protection by better household WASH, better household WASH prevalence, and ViPS vaccination against typhoid in all cluster members present at baseline using Cox proportional hazard models. Overall, ViPS vaccination was associated with a 55% (*P* < 0.001; 95% CI, 35–69) reduction of typhoid risk and was similar regardless of better WASH in the residence. Living in a better WASH household was associated with a typhoid risk reduction of 31% (*P* = 0.16; 95% CI, –16 to 59) overall. The reduction was 48% (*P* = 0.05; 95% CI, –1 to 73) in Hep A clusters, 6% (*P* = 0.85; 95% CI, –82 to 51) in ViPS clusters, and 57% (*P* < 0.05; 95% CI, 15–78) in the population during the 2 years preceding the trial. These findings demonstrate a preventive association of better household WASH in the non-ViPS population, but, unexpectedly, an absence of additional protection from ViPS by better WASH in the ViPS population. This analysis highlights the importance of assessing the combination of WASH in conjunction with typhoid vaccines, and has implications for the evaluation of new-generation typhoid conjugate vaccines.

## INTRODUCTION

Typhoid fever, an invasive bloodstream infection caused by *Salmonella enterica* serovar Typhi, remains a significant public health problem. ^1^^–^ ^4^ Globally, there are approximately 12 million cases and 128,000 deaths each year, with almost all of the burden concentrated in low- and middle-income countries. ^5^ Infection occurs through ingestion of contaminated food or water, and transmission can be high in areas with an insufficient clean water infrastructure and limited access to sanitary facilities. We know that major improvements in municipal water, sanitation, and hygiene (WASH) have drastically reduced infectious disease transmission, including typhoid, and have prevented disease in affluent countries. ^6^ Empiric evidence from a Kolkata urban slum has demonstrated that improvements in household WASH occurring naturally, without a specific intervention, were predictive of reduced typhoid risk. ^7^ Several effective typhoid vaccines are now licensed and available, including two that are WHO prequalified: a vaccine consisting of Vi polysaccharide (ViPS) and one that is ViPS conjugated chemically to a tetanus toxoid. ^8^ Recognizing that current recommendations for control of typhoid in low- and middle-income countries emphasize the use of both effective typhoid vaccines and improvement of WASH, ^9^ we asked whether protection by an effective typhoid vaccine acted independently of protection conferred by better existing WASH, and whether these independent effects were complementary when implemented together in an urban slum where typhoid is endemic. A cluster-randomized trial of an effective typhoid vaccine, ViPS, conducted in an urban slum of Kolkata, India, provided a unique platform to address these questions.

## METHODS

### Trial and population.

We conducted a cluster-randomized, controlled trial of typhoid ViPS vaccine in an urban slum in Kolkata, India, conducted between 2004 and 2006. ^10^ Demographic information and household WASH characteristics were collected from the trial population during a census conducted just before vaccination (baseline). All 62,756 individuals residing in the study area at baseline were included in the analysis. Individuals migrating into the study area and births occurring during the 2-year surveillance period were excluded.

### Vaccination.

Eighty clusters were assigned randomly to two groups of 40 clusters and received either a single-dose ViPS typhoid vaccine (Typherix, GSK, Philadelphia, PA) or a single-dose inactivated hepatitis A (Hep A) vaccine (Havrix, GSK, Philadelphia, PA). The vaccines were administered to participants who were 24 months and older. For this randomization, clusters were stratified by ward, size of population 18 years or younger, and size of population older than 18 years. Vaccination occurred between November 27 and December 31, 2004. Of 61,280 age-eligible individuals, 37,673 were vaccinated: 18,869 in the ViPS vaccine arm and 18,804 in the Hep A arm. The trial was masked at four levels: participant, care provider, investigator, and outcomes assessors.

### Clinical surveillance.

Passive surveillance for typhoid fever was conducted for 2 years after vaccination. In addition, as reported elsewhere, identical methods of demographic, WASH, and clinical surveillance were conducted in the 57,949 residents of all ages in the study area, none of whom had received either study vaccine during the 2 years before the inception of the vaccine trial. ^7^ Residents of the study area presenting to one of five study clinics with a febrile episode underwent blood culture. A febrile episode was defined as history of fever for at least three days, and sequential visits, where date of discharge and subsequent date of fever onset were separated by 14 or fewer days, were considered as a single episode. Typhoid fever was defined as a febrile episode in which *Salmonella enterica* serovar Typhi was isolated from one or more blood cultures, and a home visit conducted within 7 days after discharge confirmed that the person whose name was given at the surveillance site had indeed sought care on the date of presentation.

### Definition of WASH.

**Figure 1.**
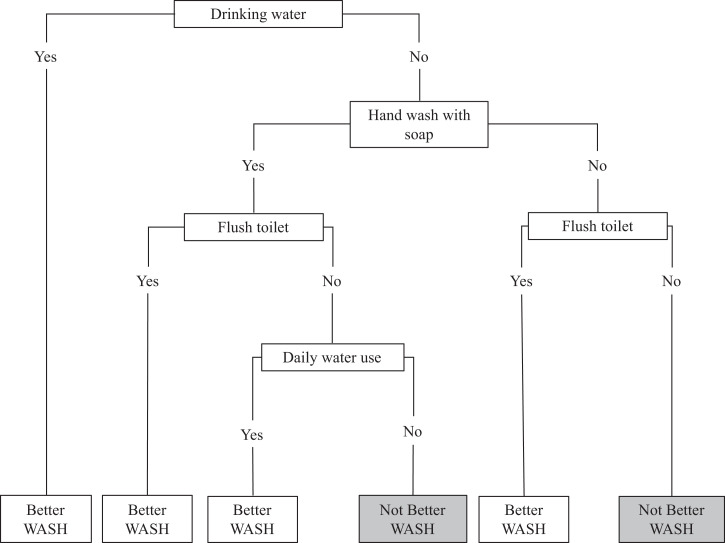
Decision tree for categorization of “better” and “not better” household water, sanitation, and hygiene (WASH) based on four dichotomous WASH variables.

As described previously, ^7^ household WASH characteristics were collected during a baseline census of the trial population. Using these data, we developed a composite dichotomous (better, not better) household WASH variable comprising several WASH features that predicted typhoid risk independent of household socioeconomic status. A better WASH household was defined based on criteria using the following variables: having a drinking water source from a private tap, well, or pump; always washing hands with soap after defecation; having a private or shared flush toilet; and using filtered or boiled water for daily household use (Figure 1).

### Analytical approach.

We evaluated the overall ViPS vaccine protection against typhoid by comparing rates of blood culture-confirmed typhoid fever among all residents, vaccinated or not, of ViPS clusters versus all residents of Hep A clusters. Correspondingly, we evaluated the protection associated with better WASH in the household by comparing rates of typhoid in all members of better WASH households versus not better WASH households. Last, we assessed the protection associated with the prevalence of better WASH households in the clusters by comparing rates of typhoid in members of households in clusters with lower prevalence versus those in clusters with higher prevalence. To explore these associations further, we undertook analyses of overall ViPS protection of individuals by better versus not better WASH of the households in which they lived, as well as by the prevalence of better WASH households of the clusters in which they resided, and evaluated evidence for a modifying effect of WASH on ViPS protection and vice versa. To evaluate even further the impact on typhoid of living in a better versus not better WASH household, as well as the impact of living in a cluster with a greater versus lesser prevalence of better WASH in the household, we examined these associations separately for the clusters assigned to ViPS and those assigned to Hep A.

### Statistical analysis.

Simple associations of typhoid risk between groups were assessed using a χ ^2^ test for heterogeneity. To measure the protection by ViPS in models, we used Cox proportional hazard regression analysis after evaluating proportionality assumptions for independent variables. Time zero at follow-up was considered the day of vaccination for vaccine recipients, and the median day of vaccination in the cluster of residence for unvaccinated persons. For individuals present at time zero, follow-up continued for 2 years until the study end date on Dec 30, 2006, the first typhoid episode, outmigration, or death, whichever occurred first. As described earlier, typhoid surveillance also was performed for the population in the trial area during the 2 years leading up to the trial. Classification of WASH of the households during this lead-in period was performed in a manner identical to that used for the trial, using information from a baseline census conducted before inception of surveillance, and the follow-up of this closed cohort was conducted in the same manner as the follow-up of subjects assembled at the onset of the trial. In individuals with multiple typhoid episodes, only the first episode was considered in the analysis. Models were adjusted for the variables used to define strata for randomization. Additional covariates (age, gender, Hindu religion, household population size, monthly household expenditures, distance to the nearest treatment center [above the median]) were selected with a forward stepwise algorithm based on a significance level of *P* < 0.05. An interaction term between household WASH and ViPS cluster assignment and good WASH prevalence and ViPS cluster assignment, respectively, was introduced into models to evaluate the presence of modifying effects. The protective effectiveness of ViPS, better WASH in the household, and prevalence of better WASH in households in the cluster was calculated as (1 – Hazard ratio) × 100, where the hazard ratio was estimated by exponentiation of the coefficient for the relevant variable in the model, and the 95% CI for the hazard ratio was estimated using a robust sandwich method to account for the design effect of cluster randomization for the trial population. The analysis was performed using the “survival” package for the Cox model, the “car” package for collinearity, and the “dplyr” package for data management in R-Studio analytical software. ^11^^–^ ^13^

## RESULTS

### Assembly of the trial population.

**Figure 2.**
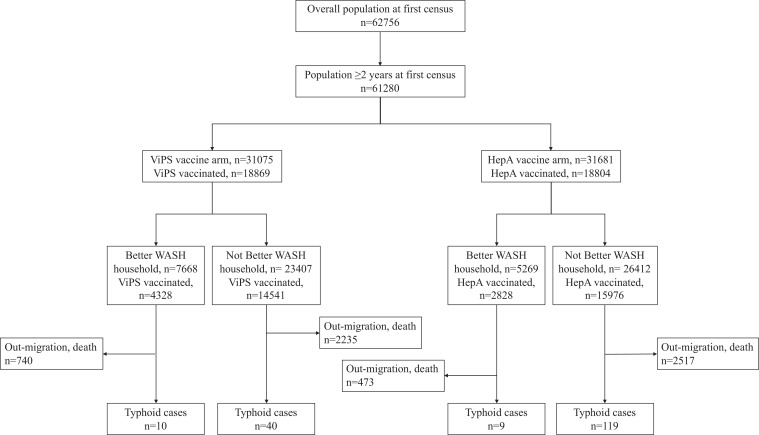
CONSORT diagram showing the population at the first census and incident typhoid cases in Vi polysaccharide (ViPS) and hepatitis A (Hep A) vaccine clusters classified by residence in better or not better water, sanitation, and hygiene (WASH) household.

In total, 62,756 individuals, of whom 61,280 were age-eligible to receive a vaccine, residing in 80 clusters, were present at the baseline census just before inception of the trial, and clusters were assigned randomly with equal probability to receive either ViPS (*n* = 31,075) or a control (*n* = 31,681) (Figure 2). There were 178 confirmed typhoid episodes recorded during follow-up, 50 cases in the ViPS clusters and 128 cases in the Hep A clusters. Within the ViPS clusters, 10 cases occurred in better WASH households and 40 cases in not better WASH households; in Hep A clusters, nine cases occurred in better WASH households and 119 cases in not better WASH households (Figure 2). With the exception of better WASH in the household, the treatment arms were well balanced with respect to the baseline characteristics, although several variables showed statistically significant differences as a result of the large sample size for the study (Table 1).

**Table 1:** Baseline comparability between Vi polysaccharide and hepatitis A clusters for analyses of overall vaccine protection by Vi polysaccharide

Variable	Hep A cluster	ViPS cluster	*P *value
Better WASH households, *n *(%)	1,044 (18)	1,455 (26)	< 0.001
Age, y at baseline; mean ± SD	28.1 ± 18.2	28.7 ± 18.3	< 0.001
Male gender, *n *(%)	17,111 (54)	16,680 (54)	0.406
Hindu religion, *n *(%)	16,878 (53)	18,794 (61)	< 0.001
Household members, mean ± SD	7.2 ± 3.8	7.3 ± 4	0.001
Expenditure,* mean ± SD	3,504.5 ± 2,432.3	3,531.1 ± 2,206.6	0.152
Treatment center distance greater than median,† m; *n* (%)	16,516 (52)	14,859 (48)	< 0.001

Hep A = hepatitis A; ViPS = Vi polysaccharide; WASH = water, sanitation, and hygiene.

* Expenditure calculated as average monthly expenditures in Indian rupees.

† Distance measured in meters as the minimum straight-line distance from a household to the closest study center.

### Overall preventive impact of residence in the ViPS vaccine clusters, member of a better WASH household, and residence in a household in clusters with greater WASH prevalence.

Vaccine coverage was 61% (18,869 of 31,075) in ViPS clusters and 59% (18,804 of 31,681) in Hep A clusters. The prevalence of better WASH households was 26% (1,455 of 5,685) in ViPS clusters and 18% (1,044 of 5,824) in Hep A clusters (Table 1). The incidence of typhoid fever in members of ViPS clusters was 0.84/1,000 person-years, and in Hep A clusters was 2.12/1,000 person-years (*P* < 0.001; Table 2). Residence in ViPS clusters was associated with an overall adjusted protective effectiveness of 55% (*P* < 0.001; 95% CI, 35–69) against typhoid (Table 2). The incidence of typhoid fever in members of better WASH households was 0.77/1,000 person-years; and in not better WASH households, the incidence was 1.67/1,000 person-years (*P* = 0.001). Residence in a better WASH household was associated with an overall adjusted protective effectiveness of 31% (*P* = 0.16; 95% CI, –16 to 59 among residents of all clusters (Table 2). Evidence for an interaction between household WASH and ViPS was not observed (*P* = 0.331).

**Table 2:** Protective effectiveness of Vi polysaccharide vaccine; better household water, sanitation, and hygiene; and better household water, sanitation, and hygiene prevalence against typhoid

	*n*	PY	Typhoid cases, *n*	IR (per 1,000 PY)	PE	
					Crude PE, % (95% CI)	Adjusted* PE, % (95% CI)
ViPS vaccine cluster	31,075	59,397	50	0.84	60%† (39–74)	55† (35–69)‡
Hep A vaccine cluster	31,681	60,387	128	2.12		
Better WASH households	12,937	24,724	19	0.77	54%§† (22–73)	31 (–16 to 59)^‖^
Not better WASH households	49,819	95,059	159	1.67		
Better WASH prevalence (lower tertile)**	20,486	39,063	81	2.07	Ref.	Ref.
Better WASH prevalence (middle tertile)**	21,164	40,306	57	1.41	32 (–17 to 60)	17 (–32 to 48)^‖^
Better WASH prevalence (upper tertile)**	21,106	40,414	40	0.99	52§ (19–72)	–19 (–96 to 27)^‖^

Hep A = hepatitis A; IR = incidence rate; PE = protective effectiveness; PY = person-years; Ref. = reference; ViPS = Vi polysaccharide; WASH = water, sanitation, and hygiene. When evaluated in models including ViPS, WASH measured as either household WASH or better WASH coverage, stratifying variables and covariates, there was no statistical evidence for an interaction between household WASH and ViPS cluster (*P *= 0.331) or between better household WASH coverage and ViPS cluster (*P *= 0.222). In models that expressed better WASH prevalence on a continuous scale, each 1% increase in better WASH prevalence was associated with a preventive impact against typhoid of 1.73% (*P *= 0.02; 95 CI, 0.32–3.12; crude) and –0.02% (*P *= 0.98; 95% CI, –1.43 to 1.37; adjusted.^‖^

* All models were adjusted for cluster stratification variables (ward [wards 29 and 30], number of residents ≤ 18 years [< 200 persons, ≥ 200 persons]; number of residents >18 years [< 500 persons, ≥ 500 persons]), design effect, and selected covariates.

† *P *< 0.001.

‡ Model adjusted for the covariates household WASH, age, Hindu religion, and longer distance to the nearest treatment center than median.

§ *P *< 0.01.

^‖ ^Model adjusted for the covariates vaccine assignment, age, Hindu religion, and longer distance to the nearest treatment center than median.

The incidence of typhoid fever in members of households in the lower tertile of better WASH household prevalence was 2.07/1,000 person-years; in the middle tertile, incidence was 1.41/1,000 person-years; and in the upper tertile, incidence was 0.99/1,000 person-years (*P* < 0.001). Relative to the lower tertile of better WASH household prevalence, residence in the middle tertile was associated with an adjusted protective effectiveness of 17% (*P* = 0.43; 95% CI, –32 to 48); residence in the upper tertile was associated with an adjusted protective effectiveness of –19% (*P* = 0.49; 95% CI, –96 to 27). In models that expressed better WASH prevalence on a continuous scale, each 1% increase in better WASH prevalence was associated with an adjusted protective effectiveness of –0.02% (*P* = 0.98; 95% CI, –1.43 to 1.37) (Table 2). Evidence for an interaction between better WASH coverage and ViPS was not observed (*P* = 0.222).

### Incidence of typhoid by vaccination and household WASH status.

Typhoid incidence was lowest in members of ViPS clusters residing in households with better WASH (0.68/1,000 person-years), highest in members of Hep A clusters residing in households with not better WASH (2·37/1,000 person-years), and intermediate in members of ViPS clusters residing in not better WASH households (0.89/1,000 person-years) and in members of Hep A clusters residing in better WASH households (0.89/1,000 person-years, *P* < 0.001 for heterogeneity of incidence in the four groups) (Tables 3 and 4).

**Table 3:** Protective effectiveness of Vi polysaccharide and better household water, sanitation, and hygiene stratified by water, sanitation, and hygiene and vaccine status

Household WASH status	ViPS cluster			Hep A cluster			ViPS PE	
	*n*	PY	Typhoid cases (IR per 1,000 PY)	*n*	PY	Typhoid (IR per 1,000 PY)	Crude PE, % (95% CI)	Adjusted* PE, % (95% CI)
Not better	23,407	44,755	40 (0.89)	26,412	50,304	119 (2.37)	62† (44–75)	61† (44–73)‡
Better	7,668	14 642	10 (0.68)	5,269	10,082	9 (0089)	23 (–108 to 72)	58§ (14–79)^‖^

Hep A = hepatitis A; IR = incidence rate; PE = protective effectiveness; PY = person-years; ViPS = Vi polysaccharide; WASH = water, sanitation, and hygiene.

* *P *< 0.01.

† *P *< 0.001.

‡ Model adjusted for the covariates age, household population size, and longer distance to the nearest treatment center than median.

§ *P *< 0.05.

^‖ ^Model adjusted for the covariates age, religion, and longer distance to the nearest treatment center than median.

**Table 4:** Protective effectiveness of Vi polysaccharide and better household water, sanitation, and hygiene stratified by water, sanitation, and hygiene and vaccine status

Better WASH PE	ViPS cluster, % (95% CI)	Hep A cluster, % (95% CI)
Crude PE	24 (–62 to 64)	62† (30–80)
Adjusted PE	6 (–82 to 51)*	48 (–1 to 73)‡

Hep A = hepatitis A; PE = protective effectiveness; ViPS = Vi polysaccharide; WASH = water, sanitation, and hygiene.

* Model adjusted for the covariates age and monthly household expenditures.

† *P *< 0.01.

‡ Model adjusted for the covariates age, household population size, and longer distance to the nearest treatment center than median.

### Protection by vaccine assignment in the cluster of residence according to better WASH in the household and prevalence of better WASH in the clusters.

In members of better WASH households, residence in ViPS clusters was associated with an adjusted protective effectiveness of 58% (*P* < 0.05; 95% CI, 14–79). Similarly, in members of not better WASH households, residence in ViPS clusters was associated with an adjusted protective effectiveness of 61% (*P* < 0.001; 95% CI, 44–73) (Tables 3 and 4). Residence in the ViPS clusters was associated with an adjusted protective effectiveness of 67% (*P* < 0.01; 95% CI, 27–85) for clusters in the lowest tertile of better WASH prevalence, 48% (*P* < 0.05; 95% CI, 6–71) in the middle tertile, and 50% (*P* < 0.05; 95% CI, 10–73) in the highest tertile (Tables 5 and 6).

**Table 5:** Protective effectiveness of Vi polysaccharide and better household water, sanitation, and hygiene prevalence stratified by water, sanitation, and hygiene prevalence tertile and vaccine status

WASH prevalence	ViPS cluster			Hep A cluster			ViPS PE	
	*n*	PY	Typhoid (IR per 1,000 PY)	*n*	PY	Typhoid (IR per 1,000 PY)	Crude PE, % (95% CI)	Adjusted PE, % (95% CI)*
Lower tertile†	7,673	14,612	9 (0.62)	12,813	24,452	72 (2.94)	79‡ (62–89)	67§ (27–85)^‖^
Middle tertile†	9,615	18,394	19 (1.03)	11,549	21,912	38 (1.73)	40 (–19 to 70)	48¶ (6–71)#
Upper tertile†	13,787	26,391	22 (0.83)	7,319	14,023	18 (1.28)	35 (–27 to 67	50¶ (10–73)#

Hep A = hepatitis A; IR = incidence rate; PE = protective effectiveness; PY = person-years; ViPS = Vi polysaccharide; WASH = water, sanitation, and hygiene. In models that expressed better WASH prevalence on a continuous scale, in the ViPS clusters, each 1% increase in better WASH prevalence was associated with a preventive impact against typhoid of –0.06% (*P* = 0.94; 95% CI, –1.59 to 1.45; crude) and –0.58% (*P* = 0.55; 95% CI, –2.50 to 1.30; adjusted).* In Hep A clusters, each 1% increase in better WASH prevalence was associated with a preventive impact against typhoid of 2.52% (*P* < 0.01; 95% CI, 0.93–408; crude) and 0.96% (*P* = 0.3; 95% CI, –1.00 to 2.88; adjusted).†

* All models were adjusted for cluster stratification variables (ward [wards 29 and 30], number of residents ≤ 18 years [< 200 persons, ≥ 200 persons]; number of residents >18 years [< 500 persons, ≥ 500 persons]), design effect, and selected covariates.

† Lower, < 6% prevalence; middle, 6% to 26% prevalence; upper, ≥ 26% prevalence.

‡ *P *< 0.001.

§ *P* < 0.01.

^‖^ Model adjusted for the covariates age, Hindu religion, household population size, and longer distance to the nearest treatment center than median.

¶ *P* < 0.05.

# Model adjusted for the covariates age, household population size, and monthly household expenditures.

**Table 6:** Protective effectiveness of Vi polysaccharide and better household water, sanitation, and hygiene prevalence stratified by water, sanitation, and hygiene prevalence tertile and vaccine status

Protective effectiveness	ViPS cluster, % (95% CI)	Hep A cluster, % (95% CI)
Middle vs. lower		
Crude PE	−68 (–231 to 15)	41 (–9 to 68)
Adjusted PE	−50 (–177 to 19)*	25 (–45% to 61)†
Upper vs. lower		
Crude PE	−35 (–175 to 33)	56 (24–75)‡
Adjusted PE	−55 (–303 to 40)*	–5 (–86 to 41)†

Hep A = hepatitis A; PE = protective effectiveness; ViPS = Vi polysaccharide; WASH = water, sanitation, and hygiene. In models that expressed better WASH prevalence on a continuous scale, in the ViPS clusters, each 1% increase in better WASH prevalence was associated with a preventive impact against typhoid of –0.06% (*P*  = 0.94; 95% CI, –1.59 to 1.45; crude) and –0.58% (*P* = 0.55; 95% CI, –2.50 to 1.30; adjusted).* In Hep A clusters, each 1% increase in better WASH prevalence was associated with a preventive impact against typhoid of 2.52% (*P* < 0.01; 95% CI, 0.93–408; crude) and 0.96% (*P* = 0.3; 95% CI, –1.00 to 2.88; adjusted).†

* Model adjusted for the covariates age and monthly household expenditures.

† Model adjusted for the covariates age, household population size, and longer distance to the nearest treatment center than median.

‡ *P* < 0.01.

### Protective impact of better WASH in the household and prevalence of better WASH in the clusters according to vaccine assignment of the cluster of residence.

In ViPS clusters, residence in a better WASH household failed to show a protective association with the risk of typhoid (adjusted protective effectiveness, 6%; *P* = 0.85; 95% CI, –82 to 51) (Tables 3 and 4). This absence of a protective association was seen both for recipients of ViPS (adjusted protective effectiveness, 16%; *P* = 0.65; 95% CI, –79 to 61) and non-recipients of ViPS (adjusted protective effectiveness, –3%; *P* = 0.96; 95% CI, –222 to 67). In contrast, in Hep A clusters, residence in a better WASH household was associated with an adjusted protective effectiveness of 48% (*P* = 0.05; 95% CI, –1 to 73) (Tables 3 and 4).

In ViPS clusters, relative to residence in the lower tertile of better WASH household prevalence, residence in the middle tertile was associated with an adjusted protective effectiveness of –50% (*P* = 0.20; 95% CI, –177 to 19), and residence in the upper tertile was associated with an adjusted protective effectiveness of –55% (*P* = 0.37; 95% CI, –303 to 40) (Tables 5 and 6). In Hep A clusters, relative to residence in the lower tertile of better WASH household prevalence, residence in the middle tertile was associated with an adjusted protective effectiveness of 25% (*P* = 0.39; 95% CI, –45 to 61), and residence in the upper tertile was associated with an adjusted protective effectiveness of –5% (*P* = 0.87; 95% CI, –86 to 41) (Tables 5 and 6). In models that expressed better WASH prevalence on a continuous scale, in ViPS clusters, each 1% increase in better WASH prevalence was associated with an adjusted protective effectiveness of –0.58% (*P* = 0.55; 95% CI, –2.50 to 1.30), whereas in Hep A clusters, each 1% increase was associated with an adjusted protective effectiveness of 0.96% (*P* = 0·33; 95% CI, –1.00 to 2.88) (Tables 5 and 6). The presence of collinearity prevented simultaneous assessment of household WASH and household WASH prevalence.

### Further evaluation of the association of better WASH in the household and typhoid risk in a population not vaccinated with ViPS.

Because the association between better WASH in household and the risk of typhoid in household residents in the Hep A clusters was of borderline statistical significance (*P* = 0.05), we assessed the relationship in the 57,949 unvaccinated residents of the study area monitored during the 2 years before the inception of the vaccine trial. These residents were assessed with the same procedures and with the same data collection instruments (including the same definition of better WASH in the household) as those used during the trial. During this lead-in period, the incidence of typhoid fever in members of better WASH households was 0.46/1,000 person-years; in not better households, incidence was 1.36/1,000 person-years (*P* < 0.05). After adjusting for potential confounding variables, a 57% reduction of typhoid risk was observed in residents of households with better WASH compared with residents in households with not better WASH (*P* < 0.05; 95% CI, 15–78).

## DISCUSSION

In this trial, living in a cluster vaccinated with ViPS was associated with protection against typhoid, and the level of protection was similar regardless of whether the individual lived in a household with better WASH. Conversely, living in a better WASH household was associated with suggestively reduced typhoid risk among members of all the clusters, but this reduction was only evident in clusters that received Hep A—a finding consistent with protection observed in members of households with better WASH in the population monitored for the 2 years of surveillance preceding the trial. A greater prevalence of better WASH households in the cluster was not associated with a reduced typhoid risk overall in all clusters, nor was it seen in the ViPS and Hep A clusters considered separately.

Before discussing the implications of these findings, it is important to consider the limitations of our study. First, our variable classifying households as having better or not better WASH was constructed using simple variables collected at baseline, which may not have fully captured or might not have served as proxies for the WASH behaviors and facilities responsible for the reduction of risk of typhoid in the household. As a result, our household WASH classification variable may have underestimated the true impact of household WASH against typhoid. Second, our definition of better WASH may be idiosyncratic to the population under study and may not be generalizable to other settings. Nonetheless, our findings support the concept that better WASH in the household occurring spontaneously without external intervention was associated with a reduced risk of typhoid in the slums participating in the trial. Third, although the trial cluster-randomized vaccination, it did not randomize WASH. However, we controlled for potentially confounding variables in our analyses. Fourth, because of the collinearity of household-level WASH and neighborhood WASH prevalence, it was not possible to evaluate the independent protection associated with these two variables when both were considered simultaneously in our analyses. Last, our findings, although elucidating the separate and joint impacts of ViPS and WASH in the setting for the study, may or may not apply to other settings that have different WASH practices or to different typhoid vaccines.

Our analyses also have several important strengths. The analyses were conducted on data collected in the context of a prospective cluster-randomized trial of a well-defined population under comprehensive follow-up for typhoid. It used a previously developed and validated definition of better household WASH. In addition, typhoid was detected with clear clinical criteria, including blood culture confirmation, and diagnoses were made without knowledge of either vaccination or household WASH status.

Our analyses produced two unexpected observations. First, the protective association of better household WASH was absent in clusters assigned to ViPS, suggesting no additional benefit of WASH in a vaccinated population. That living in a household with better WASH was not associated with a lower risk of typhoid in clusters targeted for ViPS, but was in those targeted for Hep A, might be related to the observation that ViPS was shown earlier to confer substantial herd protection in this trial, reflecting a reduction of the intensity of typhoid transmission in the ViPS clusters. ^10^ It is possible that the reduction of typhoid transmission in the better WASH households resulted in less of a reduction of typhoid disease incidence in the lower transmission settings of the ViPS clusters, as evidenced by the shape of the curve relating the size of the ingested inoculum of typhoid and the risk of typhoid disease. ^14^ However, because the interaction term between ViPS and WASH variables in this analysis was not significant, lack of statistical heterogeneity indicates that apparent differences in typhoid risk in the vaccine groups may rather be a result of random variation and small sample size, and not the modification of WASH effects by vaccination status. Second, protection associated with ViPS was similar regardless of household WASH status. We anticipated that the protective association of better household WASH, mediated by reducing exposure to the infectious agent within the household, would enhance the protective effects of the vaccine. However, this observation may be consistent with the similar levels of ViPS protection recorded in randomized trials done in Nepal, South Africa, and China ^15^^–^ ^17^—sites with different rates of typhoid infection, possibly mimicking settings where exposure to typhoid varies as a result of the presence or absence of improved WASH.

In aggregate, our findings suggest that minor improvements in WASH, which already existed within this poor urban slum study setting and are therefore affordable and attainable, were associated with a substantial reduction in the risk of typhoid in the population that had not received an effective typhoid vaccine. However, these improvements failed to reduce further the risk of typhoid in a population that had received an effective typhoid vaccine. Although our observations do not argue against possible combined protection against endemic typhoid by well-designed WASH interventions and effective typhoid vaccines, our data do not support the assertion that naturally occurring improvements of household WASH added to the protection by ViPS vaccine in this urban slum setting. Although ViPS is no longer the primary recommended vaccine for developing countries, our unexpected results underscore the importance of evaluating the combined and independent effects of WASH and effective typhoid vaccines—in particular, Vi conjugate vaccines—which are now approved and recommended for use in endemic settings.
